# Using Wearables to Study Biopsychosocial Dynamics in Couples Who Cope With a Chronic Health Condition: Ambulatory Assessment Study

**DOI:** 10.2196/49576

**Published:** 2024-08-05

**Authors:** Theresa Pauly, Janina Lüscher, Lea Olivia Wilhelm, Melanie Alexandra Amrein, George Boateng, Tobias Kowatsch, Elgar Fleisch, Guy Bodenmann, Urte Scholz

**Affiliations:** 1 Department of Gerontology Simon Fraser University Vancouver, BC Canada; 2 Swiss Paraplegic Research Nottwil Switzerland; 3 Faculty of Health Sciences and Medicine University of Lucerne Lucerne Switzerland; 4 Medical School Berlin Berlin Germany; 5 Department of Education and Psychology Freie Universität Berlin Berlin Germany; 6 Department of Health Sciences Helsana Group Zurich Switzerland; 7 Centre for Digital Health Interventions Department of Management, Technology, and Economics ETH Zurich Zurich Switzerland; 8 Institute for Implementation Science in Health Care University of Zurich Zurich Switzerland; 9 School of Medicine University of St. Gallen St.Gallen Switzerland; 10 Institute of Technology Management University of St.Gallen St. Gallen Switzerland; 11 Department of Psychology University of Zurich Zurich Switzerland; 12 University Research Priority Area Dynamics of Healthy Aging University of Zurich Zurich Switzerland

**Keywords:** couples, wearables, type II diabetes, heart rate, biopsychosocial dynamics, physiological linkage, mobile health, technology, social support, chronic disease, usability, utility, mHealth

## Abstract

**Background:**

Technology has become an integral part of our everyday life, and its use to manage and study health is no exception. Romantic partners play a critical role in managing chronic health conditions as they tend to be a primary source of support.

**Objective:**

This study tests the feasibility of using commercial wearables to monitor couples’ unique way of communicating and supporting each other and documents the physiological correlates of interpersonal dynamics (ie, heart rate linkage).

**Methods:**

We analyzed 617 audio recordings of 5-minute duration (384 with concurrent heart rate data) and 527 brief self-reports collected from 11 couples in which 1 partner had type II diabetes during the course of their typical daily lives. Audio data were coded by trained raters for social support. The extent to which heart rate fluctuations were linked among couples was quantified using cross-correlations. Random-intercept multilevel models explored whether cross-correlations might differ by social contexts and exchanges.

**Results:**

Sixty percent of audio recordings captured speech between partners and partners reported personal contact with each other in 75% of self-reports. Based on the coding, social support was found in 6% of recordings, whereas at least 1 partner self-reported social support about half the time (53%). Couples, on average, showed small to moderate interconnections in their heart rate fluctuations (*r*=0.04-0.22). Couples also varied in the extent to which there was lagged linkage, that is, meaning that changes in one partner’s heart rate tended to precede changes in the other partner’s heart rate. Exploratory analyses showed that heart rate linkage was stronger (1) in rater-coded partner conversations (vs moments of no rater-coded partner conversations: *r*_diff_=0.13; *P*=.03), (2) when partners self-reported interpersonal contact (vs moments of no self-reported interpersonal contact: *r*_diff_=0.20; *P*<.001), and (3) when partners self-reported social support exchanges (vs moments of no self-reported social support exchange: *r*_diff_=0.15; *P*=.004).

**Conclusions:**

Our study provides initial evidence for the utility of using wearables to collect biopsychosocial data in couples managing a chronic health condition in daily life. Specifically, heart rate linkage might play a role in fostering chronic disease management as a couple. Insights from collecting such data could inform future technology interventions to promote healthy lifestyle engagement and adaptive chronic disease management.

**International Registered Report Identifier (IRRID):**

RR2-10.2196/13685

## Introduction

Coping with a chronic health condition refers to the various psychological, emotional, and behavioral strategies individuals use to manage the challenges, stressors, and lifestyle adjustments associated with living with a long-term health condition [1]. Challenges associated with the maintenance of health-promoting behaviors such as being physically active, following a medication regime, and monitoring one’s current health status on top of direct effects of the chronic health condition on cognitive and physical resources (eg, fatigue) can lead to lapses in treatment adherence, which are common [2]. Poor treatment adherence, in turn, results in higher morbidity and mortality rates, as well as increased expenses related to outpatient care and hospitalization for managing diabetes-related complications [3]. Models of chronic disease management acknowledge the important role that close others generally, and romantic partners specifically, play in coping [1,4-6]. Social support refers to how social relationships provide resources and assistance to manage stressors and challenges [7]. These forms of assistance manifest in instrumental ways (ie, practical assistance with a problem or task) and emotional ways (ie, comfort, encouragement, reassurance, and listening empathetically [8]). Social support can facilitate coping by promoting healthy lifestyle choices and helping manage disease-related demands in daily life and has been associated with better disease adjustment and higher quality of life among patients [9,10]. Moreover, in a dyadic context, providing social support has also been conceptualized as a specific form of coping [11]. In laboratory stress paradigms, social support (eg, in the form of physical touch) buffers physiological stress reactivity and speeds up recovery, for example, as assessed by heart rate and salivary cortisol levels [12,13]. This effect is particularly pronounced when the social support comes from a close person, such as a romantic partner [12].

The couple as a unit of study is particularly interesting for researchers studying chronic health conditions because romantic partners tend to be a major source of support and partners’ health is closely linked [11,14,15]. For example, individuals are more than 2 times more likely to have diabetes themselves when they are in a relationship with someone who has diabetes [16,17]. This is not surprising as lifestyle risk factors for diabetes such as eating patterns, physical activity, and other health behaviors are often shared in couples [18]. Newer conceptual models of chronic health conditions and coping, including the dyadic regulation connectivity model [5], see couples as a dynamic system, in which dyadic regulation of chronic health conditions occurs in a flexible, dynamic, and complex way adjusting to changing internal contexts (eg, emotional states) as well as external contexts (eg, work demands). Specifically, dyadic regulation involves different network hubs (illness representations hub, coping behaviors hub, and outcomes hub), interconnected through feedback loops and constantly interacting at the person and dyad level. This dynamic process is described as “a ‘living’ mechanism which is continuously constructed and reconstructed in the mind and the behaviors of the individuals and the couples” [5]. Consequently, the field has called for more advanced methods to be able to study time- and context-sensitive processes shaping health behavior and disease management [19].

New technology including smartphones and smartwatches allows to collect observational and physiological data to observe dynamic phenomena as they unfold in daily life [20]. For example, researchers have equipped couples with audio recorders to capture sound snippets as they go about their usual routines, finding that use of certain speech (eg, positive word use, deep conversation) was associated with better disease adjustment to breast cancer in women [21,22]. Furthermore, health behaviors such as physical activity and medication adherence can be objectively tracked and this information can be used to develop targeted interventions in real time [23]. Researchers have also used sensor data to measure individuals’ physiological functioning, for instance, electrodermal activity (EDA), skin temperature, respiratory rate, blood pressure, and heart rate [24,25]. Measures of the autonomic nervous system such as heart rate hold particular significance because they tend to change rapidly in response to shifting contexts (eg, emotions or behavior [26]). This responsiveness, in combination with the relative ease of data collection through wearable devices, makes it an excellent indicator of physiological linkage in couples.

Studies analyzing physiological time series data in couples have found that romantic partners are interconnected in daily fluctuations of heart rate, a phenomenon that has been called “physiological synchrony” or “physiological linkage” [27-29]. Physiological linkage has been associated with central interpersonal outcomes such as trust, empathy, and effective cooperation [28,30-32], and could thus be important for social processes such as supportive interactions in managing a chronic health condition. For example, synchrony in skin conductance levels predicted higher cooperative success in 76 dyads (college students) playing a Prisoner’s Dilemma game [30]. In addition, heart rate linkage was increased in 110 college student dyads when randomized to playing a trust-related game versus a control condition [32]. A recent meta-analysis of 60 published and unpublished experiments also demonstrated that synchrony exhibits a medium-sized effect on prosocial attitudes and behaviors [33]. Thus, physiological linkage might be relevant for coregulation between partners and enable adaptive couple functioning [34]. Yet, no prior research has examined heart rate linkage in the context of everyday social contexts and social support exchanges in individuals with chronic health conditions.

Previous studies on interpersonal dynamics and heart rate linkage in couples have mostly used electrocardiogram data from medical-grade devices such as the BIOPAC collected in laboratory settings [34,35]. However, commercial devices such as smartwatches could be a cost-effective way to collect biopsychosocial data on couples managing chronic health conditions in daily life. Therefore, the objective of this study is to outline the methods we used to gather two types of data: (1) information concerning psychosocial processes, encompassing social contexts and interactions, using observational techniques (audio recordings), and self-report measures; and (2) data on biological processes, specifically heart rate. In doing so, we summarize the feasibility of collecting such data and present descriptive and exploratory findings. Specifically, we examine whether heart rate linkage in couples is higher in moments of personal contact and support.

## Methods

### Participants and Procedure

We used data of 11 couples who took part in the DYadic MANagement of Diabetes (DYMAND) study (study protocol [36]; description of monitoring system [37]). This study was funded by the Swiss National Science Foundation (CR12I1_166348). Couples were recruited through newspaper advertisement, flyer distribution to diabetes specialists and pharmacies, diabetes forums, and in diabetes departments of hospitals in the German-speaking part of Switzerland from 2019 to 2021. The original recruitment target was 180 couples. However, due to the COVID-19 pandemic and associated restrictions regarding in-person research with vulnerable populations recruitment had to be paused for a considerable duration. This resulted in the current sample size. Eligibility criteria comprised one partner having a medical diagnosis of type II diabetes with prescribed oral drugs and the other partner (without such diagnosis) being willing to participate as well. Participants were excluded if they required insulin injections, inpatient treatment, were working in shiftwork, or had insufficient command of the German language. We collected data from 13 couples, who were living in metropolitan and rural regions in the German-speaking part of Switzerland. Two couples dropped out during the monitoring phase because of time constraints, resulting in a final sample of 11 couples (see Table 1 for descriptives). Participants with type II diabetes were aged 52-80 years (mean 68.9, SD 7.7 years), mostly male (10 males, 1 female), and a majority did not have a higher secondary school degree (8 with lower secondary education, 3 with higher secondary degree). Less than half of the participants with type II diabetes measured their blood sugar levels daily (4/11, 36%) and the average long-term blood sugar concentration was 6.8% (hemoglobin A_1c_ [HbA_1c_], SD 0.5%; normal HbA_1c_ <5.7% [38]). Most participants reported a positive influence of their partnership on their diabetes management (10/11, 91%). Partners were aged 47-80 (mean 66.7, SD 9.2) years, mostly female (1 male, 10 females), and 1 participant had a higher secondary school degree. Less than half of the participants with type II diabetes (3/11, 27%) and partners (4/11, 36%) were employed, whereas the majority was retired. Couples’ average relationship duration was 31.5 (SD 14.6) years. Most couples had a monthly household income above the poverty line for a household of 2 people in Switzerland (>4000 Swiss Francs; 80% [39]).

**Table 1:** Sample descriptives (N=11 couples).

Variable	Person with type II diabetes	Partner
Age (years), mean (SD)	68.91 (7.71)	66.68 (9.19)
Sex	91% male, 9% female	9% male, 91% female
Education	73% with lower secondary degree	91% with lower secondary degree
Employment status	27% employed	36% employed
Relationship duration (years), mean (SD)	31.58 (14.95)	31.45 (15.01)
Household income (Swiss Francs^a^)	2001-4000 (n=1); 4001-6000 (n=2); 6001-8000 (n=4); 8001-10,000 (n=1); and >10,000 (n=3)	4001-6000 (n=3); 6001-8000 (n=2); 8001-10,000 (n=1); >10,000 (n=3); and missing (n=2)
Children, n	No children (n=2), 1 child (n=3), 2 children (n=3), 3 children (n=1), and 4 children (n=2)	No children (n=3), 1 child (n=1), 2 children (n=5), and 3 children (n=2)

^a^1 Swiss Franc=US $1.13.

After a baseline session in which the participants were trained to use the equipment and completed a questionnaire battery, the participants entered a 7-day monitoring phase. An assessment was triggered every hour during a specified time defined by the couples in the morning (eg, 6 AM to 9 AM) and evening (eg, 5 PM to 9 PM) during the week and all day (waking hours, eg, 8 AM to 9 PM) on the weekend. This decision was made to alleviate participant burden by abstaining from data collection during periods when partners are typically at work, thus ensuring that we capture meaningful partner interactions effectively. Assessments were elicited when partners were close to each other (when the smartwatch Bluetooth system detected a signal strength of the partner’s watch greater than –80 dB corresponding to approximately 5 m), and speech was detected. When the 2 conditions of physical closeness and speech were not detected, an assessment was triggered at the end of the hour to ensure sufficient data coverage. The assessment included a 5-minute audio and heart rate recording, followed by a brief self-report questionnaire on the smartphone which asked about partner interactions and health behaviors. Each evening, partners answered a longer survey about their own and their partner’s behaviors (data not used in this study). Participants reported that the study app was easy to use (persons with type II diabetes: mean 5.9, SD 0.7; partners: mean 6.0, SD 0.7; 1=completely disagree to 7=fully agree). See the study by Boateng et al [37] for a detailed description of the development and deployment of the smartwatch- and smartphone-monitoring system. After the 7-day monitoring phase, partners returned to the laboratory for an exit session during which feedback on the study was collected, and partners were videotaped while having a 10-minute conversation about their diabetes management. In this exit session, the participants were also allowed to review their audio recordings and delete files before the research team accessed them. None of the couples chose to remove any of the audio recordings. Couples received 100 Swiss Francs as reimbursement for taking part in the study.

### Ethical Considerations

Ethics approval was granted by the cantonal ethics committee of the Canton of Zurich, Switzerland (Req-2017_00430), and informed consent was obtained from all participants.

### Measures

#### Audio Recordings

A smartwatch (Polar M600) recorded daily life audio snippets for 5 minutes each. Four trained research assistants used the Social Environment Coding of Sound Inventory [40] to code couples’ location, activity, conversation partner, and conversation type. Conversation types included (1) practical: pragmatic conversations focusing on practical daily matters such as making plans and discussing meals; (2) small talk: an interaction without instrumental purpose, involving superficial exchanges of information that have no significant impact or consequence on participants’ lives; (3) deep or substantive: conversations with the purpose to exchange thoughts, information, values, and ideas on nonemotional topics such as current events; (4) disclosure: conversations that involve sharing personal feelings or emotions, which may include discussing topics such as the relationship, hopes and dreams, or other deeply meaningful experiences, surpassing the threshold of triviality. Furthermore, we coded for instrumental and emotional social support, following procedures outlined in the study by Wang and Repetti [41]. Instrumental support entailed help with practical problems and tasks, such as assistance with chores or the provision of information to help handle a task-oriented problem (eg, figuring out the fastest driving route to the doctor’s office). Emotional support included provisions of comfort, encouragement, advice, or guidance of an emotional nature, for example, listening empathetically to a spouse’s frustrations about work. Fifteen percent of recordings were coded by all 4 research assistants to calculate interrater reliability (intraclass correlation [ICC] of 2.1, calculated using SPSS; IBM Corp), which was satisfactory (>0.60 [42]; location: ICC=0.77, activity: ICC=0.67, conversation partners: ICC=0.60-0.74, and support: ICC=0.63), except for conversation types (ICC=0.39 for practical, 0.50 for small talk, 0.24 for deep or substantive, and 0.28 for disclosure).

#### Heart Rate

The smartwatch also collected heart rate information during the 5-minute audio recordings. The watch did not collect equally spaced recordings (mean spacing=2.7, SD 2.8 seconds). Thus, to match partner heart rate data, we calculated the mean of all heart rate values captured in 5 seconds frames (average n=2.1, SD 1.2, range 1-6 values; if aggregated to 10-second frames, pattern of findings remains the same). This resulted in a total of 55,175 heart rate values. We then aligned the heart rate data of couples by time stamp and kept only segments in which data from both partners were available (n=37,834, 69% heart rate pairings). Missing values in paired data are due to devices eliciting recordings with a small lag in partners or additional recordings that were triggered in just 1 partner. Furthermore, 5-minute units with less than 10 paired heart rate values of partners were deleted (n=6, <1%).

#### Self-Reported Social Contexts and Exchanges

Subsequently to the audio and heart rate recording, participants reported whether they had any personal contact with their partner in the last 5 minutes. Furthermore, participants with type II diabetes indicated whether they received emotional or practical support from their partner in the last 5 minutes with yes or no: “My partner has supported me emotionally in the last five minutes”/“My partner has supported me practically in the last five minutes.” Partners reported emotional and practical support provision with yes or no: “I have been emotionally supportive of my partner in the last five minutes”/“I have been practically supporting my partner in the last five minutes.” A social support exchange was coded when provided or received support was indicated by at least 1 partner.

### Statistical Analysis

Descriptives were calculated using frequencies, means, and SDs. Heart rate linkage in couples was quantified using cross-correlations [43], calculated with the ccf function in R (Stats Package; R Core Team [44]). The linkage between partners can be in-phase (positive correlation), meaning that fluctuations in partners’ heart rates are in the same direction, or antiphase (negative correlation), meaning that couples show opposite patterns of heart rate fluctuations over time. The cross-correlation models the relationship between both partners’ heart rate time series data for a given recording. An advantage of the cross-correlation method over traditional correlation techniques is that it calculates the correlation between dyadic time series of heart rate data at a given maximum lag. Specifically, it quantifies the dependence of the heart rate time series data of the person with type II diabetes on its past observations, the partner’s concurrent heart rate, and the past or future observations of the partner’s heart rate. In this way, the ccf identifies the maximum cross-correlation for each recording, which could occur at a positive, no, or negative lag. We considered only a lag of ±1 time fragment because we had averaged heart rate recordings across 5-second windows, and cross-correlations can be biased if longer lags are used (see suggestions outlined in the study by Behrens et al [45]). In the presented results, a lag of –1 indicates that changes in the heart rate of the person with type II diabetes tended to be a precursor to changes in the partner’s heart rate. A lag of 0 indicates that changes in heart rate tended to co-occur. A lag of +1 indicates that changes in the partner’s heart rate tended to be a precursor to changes in the heart rate of the person with type II diabetes. Finally, we explored whether cross-correlations might differ by social contexts and exchanges using simple random-intercept multilevel models (observations nested within individuals nested within couples, controlling for person-level averages; R package lme4 [46]). For these models, cross-correlations were Fisher-Z transformed to normalize the distribution.

## Results

### Descriptives

We collected 992 audio files from 11 couples, of which 375 captured the same content (ie, they co-occurred in partners; 37.8%). After removing the audio file of 1 partner in these cases, we had a total of 617 audio recordings for analysis (mean 56.1, SD 14.5 per couple; range: 35-83 files). Three-quarters of the audio files contained speech (74%), and participants were mostly at home (80%; 8% in public, 5% in transit, 4% outdoors, and 3% other or unknown). Participants were most frequently watching TV or listening to radio (195/590, 33%), socializing (189/590, 32%), or doing housework (69/590, 12%), and, to a lesser extent, were eating or drinking (32/590, 5%) or physically active (12/590, 2%). In 16% (85/590) of recordings, activities were unknown. Recorded conversations (423/617) mainly included partners talking (372/423, 88%), whereas conversations with friends (77/423, 18%), strangers (17/423, 4%), children (10/423, 2%), and other family members (9/423, 2%) or self-talk (24/423, 6%) occurred less frequently. Concerning conversation types, most conversations were substantive (194/423, 46%), with fewer conversations of practical content (139/423, 33%) or small talk (76/423, 18%; 3% other). Social support was coded in 6% of recorded conversations (27/423), with the most frequent support providers being the partner (16/27, 60%) or a friend (9/27, 33%). The nature of the supportive interaction was 63% (17/27) instrumental and 37% (10/27) emotional (single choice only).

We collected 606 brief self-report questionnaires from partners, of which 79 captured the same situation (completed simultaneously by the partners). Thus, we analyzed 527 self-report questionnaires. In three-quarters of the questionnaires, partners reported having had personal contact with each other (394/527, 75%). Support was self-reported in more than half of the instances (277/527, 53%), with emotional support occurring 84% (233/277) of the time and practical support occurring 82% (227/277) of the time (multiple-choice possible).

### Heart Rate Linkage

**Figure 1:**
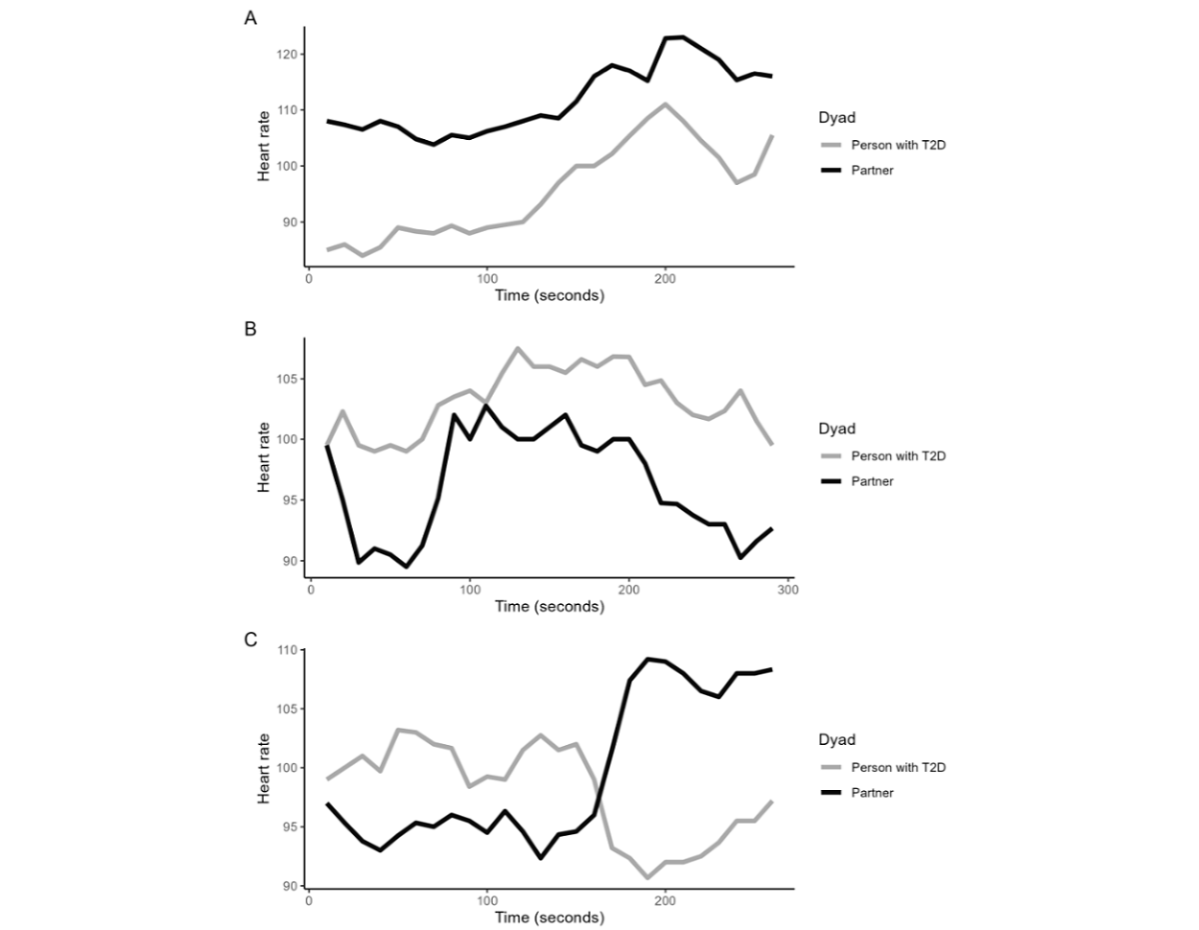
Three examples of collected dyadic heart rate data across a 5-minute recording. Panel A shows a high positive cross-correlation at a lag of 0 (*r*=0.91), indicating that partners show linked concurrent increases and decreases in heart rate in the same direction. Panel B shows a high positive cross-correlation at a lag of 1 (*r*=0.79), indicating that partners show linked increases and decreases in heart rate with heart rate changes in the partner preceding heart rate changes in the individual with type II diabetes by 5 seconds. Panel C shows a high negative cross-correlation at a lag of 0 (*r*=–0.79), indicating that partners show concurrent increases and decreases in heart rate in opposite directions (eg, one partner shows an increasing heart rate while the other shows decreasing heart rate). T2D: type II diabetes.

**Figure 2:**
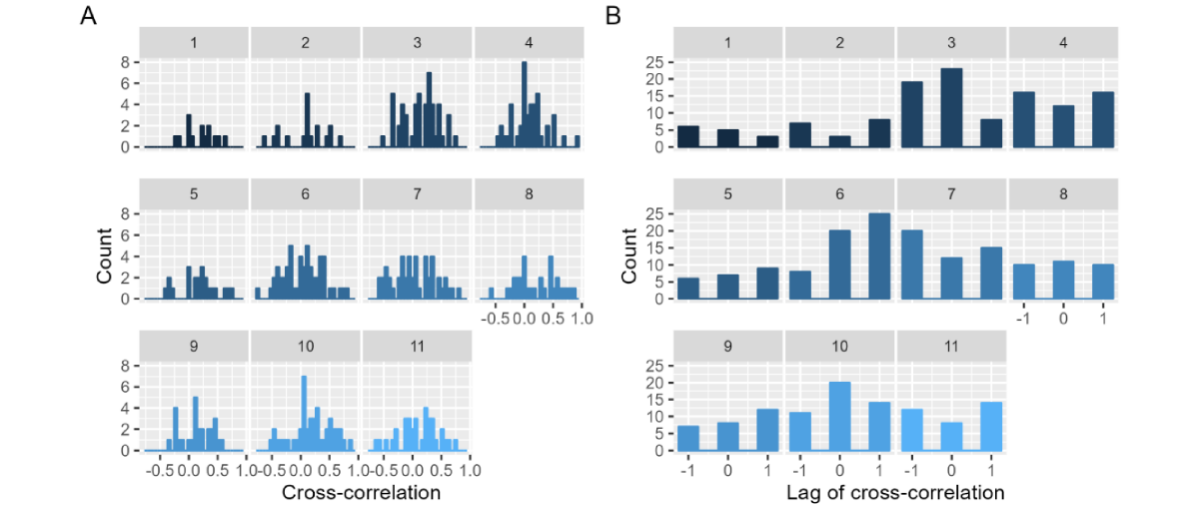
Distributions of cross-correlations (A) and the respective lags of cross-correlations (B) per couple. A lag of –1 indicates that changes in the heart rate of the person with type II diabetes tend to be a precursor to changes in the partner’s heart rate. A lag of 0 indicates that changes in heart rate tended to co-occur. A lag of 1 indicates that changes in the partner’s heart rate tend to be a precursor to changes in the heart rate of the person with type II diabetes.

Dyadic heart rate data were available for 384 of the 617 audio recordings (ie, the 5-minute segments contained heart rate data from both partners). Concerning heart rate linkage, on average, all couples showed small to moderate cross-correlations in their heart rate (range: *r*=0.04-0.22; see Table 2). However, there was considerable variation in the observed cross-correlation between recordings (SDs 0.26-0.38). This means that couples showed a large variety of cross-correlations between recordings: Each couple sometimes showed a positive cross-correlation (ie, heart rate increases in one partner were linked with heart rate increases in the other partner; see Figure 1 panels A and B for examples), sometimes a cross-correlation close to 0 (ie, changes in heart rate throughout the recording were not systematically linked), and sometimes a negative cross-correlation (eg, heart rate increases in one partner were linked with heart rate decreases in the other partner; see Figure 1 panel C for an example). Histograms of couples’ cross-correlations can be found in Figure 2, panel A.

Table 2 also denotes the average of the lags corresponding to each cross-correlation separately by couple. As outlined in the Methods section, the maximum cross-correlation could occur at a lag of +1, 0, or –1 for each recording. As seen in Figure 2 panel B, couples showed large heterogeneity in the occurrence of lags. In some couples, the extent to which heart rate changes in either the person with type II diabetes or the partner tended to precede heart rate changes in the other dyad member was balanced (couples 2, 4, 8, 10, and 11). However, in couples 1 and 7, a large share of recordings featured a negative lag (43%), suggesting that heart rate linkage in the dyad tended to be driven by the partner’s heart rate. There was also variation in the extent to which recordings occurred that featured a lag of 0 (indicating same-time linkage with no partner driving the changes)—from every fifth (17%) recording in couple 2 to almost half of recordings in couple 3 (46%).

**Table 2:** Overview of cross-correlations and their lags by couple.

Couple ID	Number of observations	Cross-correlation, mean (SD)	Lag^a^, mean (SD)	Percent negative lag	Percent no lag	Percent positive lag
1	14	0.184 (0.27)	–0.214 (0.80)	43	36	21
2	18	0.086 (0.38)	0.056 (0.94)	39	17	44
3	50	0.133 (0.30)	–0.220 (0.71)	38	46	16
4	44	0.084 (0.29)	0.000 (0.86)	36	27	36
5	22	0.172 (0.32)	0.136 (0.83)	27	32	41
6	53	0.045 (0.35)	0.321 (0.73)	15	38	47
7	47	0.036 (0.37)	–0.106 (0.87)	43	26	32
8	31	0.215 (0.37)	0.000 (0.82)	32	35	32
9	27	0.146 (0.26)	0.185 (0.83)	26	30	44
10	45	0.198 (0.34)	0.067 (0.75)	24	44	31
11	34	0.102 (0.34)	0.059 (0.89)	35	24	41

^a^A negative lag indicates that changes in the heart rate of the person with type II diabetes tend to be a precursor to changes in the partner’s heart rate. A lag of 0 indicates that changes in heart rate tended to co-occur. A positive lag indicates that changes in the partner’s heart rate tend to be a precursor to changes in the heart rate of the person with type II diabetes.

### Heart Rate Linkage and Social Contexts and Exchanges

Finally, we explored whether heart rate linkage between partners might differ by social contexts and exchanges. Using the rater codings, we found that heart rate linkage was higher in moments when partners were talking with each other (mean *r*=0.17) than in moments when they were not engaged in conversation (mean *r*=0.04; *b*=0.15, SE=0.07; *P*=.03). Self-report data replicated these findings, showing that heart rate linkage was higher in moments when participants reported having had personal contact with each other (mean *r*=0.20) than in moments when they reported no such interpersonal contact (mean *r*<0.01; *b*=0.22, SE=0.06; *P*<.001). Furthermore, heart rate linkage was higher when participants self-reported giving or receiving support (mean *r*=0.22) than in moments of no support (mean *r*=0.07; *b*=0.18, SE=0.06; *P*=.004). We did not examine associations between rater-coded social support and heart rate linkage due to its very low frequency. The findings are displayed in Multimedia Appendix 1.

## Discussion

### Principal Findings

Most chronic health conditions require major modification of one’s lifestyle, including adhering to sometimes complex medication regimes and engaging in health-promoting behaviors. Using new technology to monitor everyday biopsychosocial dynamics in couples coping with chronic health conditions could provide important insights for future intervention development. This study focused on testing a monitoring system that collected audio recordings, brief self-reports, and physiological data (heart rate) to log naturally occurring social interactions and to quantify the extent of physiological linkage in 11 persons with type II diabetes and their partners. Specifically, we used smartwatches to assess the feasibility of using commercially available devices. The smartwatches elicited 5-minute audio and heart rate recordings once an hour (mornings and evenings on weekdays, all day on weekends) when the Bluetooth signal indicated that partners were physically close, and speech was detected or when these 2 conditions were not detected, an assessment was triggered at the end of the hour to ensure sufficient data coverage. Self-reports were triggered consequently on linked smartphones to these recordings. Whereas raters coded social support as relatively infrequent (6% of audio recordings), participants self-reported that social support exchanges took place 53% of the time. On average, couples showed heart rate linkage of small to moderate degree. Notably, there was considerable variation in the extent of heart rate linkage and the occurrence of lags (indicating that one partner tended to drive heart rate changes in the other partner) across recordings within each couple. According to exploratory analyses, heart rate linkage was stronger when raters coded partner conversations and when interpersonal contact or social support exchanges were self-reported by partners. The following outlines important implications and considerations when collecting such data.

### Rater-Coded and Self-Reported Social Contexts and Exchanges

Raters coded conversations between partners in 6 out of 10 audio recordings, and couples self-reported personal contact with each other 75% of the time, speaking to a good performance of the triggering system [37]. Concerning social exchanges, raters coded social support in 6% of audio recordings, whereas partners self-reported social support in 53% of self-reports. The low frequency of rater-coded social support dovetails with a previous study showing that support occurred only in about 4% of video data captured during 4 days of healthy couples’ daily lives [41].

Self-report measures of social support may be influenced by social desirability bias and gender stereotypes [47]. For example, while self-report data support the gender support-gap hypothesis with men receiving more support from women within couples, naturalistic observational studies show that men provided the same or more everyday support to their partners than women did [41,48], although the quality of the support might differ [49]. Women might also more frequently seek support from their partners, demonstrating the importance of collecting data on support solicitation in daily life [41]. On the other hand, observational measures are less vulnerable to demand characteristics and can capture supportive interactions that are not explicitly recognized or acknowledged by the individual receiving it [40,41].

However, self-report data capture types of interactions the individual perceives as supportive, whereas such interactions may not be immediately apparent to outside observers unfamiliar with the couple’s unique dynamics. Indeed, the same action might be perceived as supportive by one person and as controlling or intrusive by another. The discrepancy between self-reported and rater-coded social support in this study emphasizes the need for a comprehensive approach to assessing social support in future research. For example, in future investigations, researchers could use text message content analysis [50] to capture conversations, expressions of empathy, encouragement, and tangible assistance between the couple. Participants could also be asked to press a button on a smartwatch whenever they are about to provide support, thereby eliciting an audio recording of the real-time support exchange. Furthermore, approaches could be developed using machine learning to automatically detect and capture moments when social support is occurring in daily life. Supplementary subjective measures could then be used to assess individuals’ satisfaction with and the quality of support they receive within these objectively captured interactions.

### Using Commercial Smartwatches to Monitor Heart Rate Linkage

Over the last decade, researchers have used different physiological parameters to demonstrate physiological linkage in couples, such as breathing rate, EDA, and cortisol [43,51,52] or voice stress [53]. With this project, we build on and extend this work by taking the research out of the laboratory and into daily life, using commercially available devices for heart rate monitoring. We found that everyday heart rate fluctuations in persons with type II diabetes and their partners showed intercorrelations of small to moderate degrees. Using an optical sensor (photoplethysmography) instead of electrical signals (electrocardiogram [ECG]) to estimate heart rate with commercially available devices has the benefit of being relatively unobtrusive, user-friendly, and affordable [54]. Contrasting consumer-grade wearables with the gold standard ECG, initial research overall shows promising findings regarding the accuracy of wrist-worn photoplethysmography devices, although they tend to underestimate heart rate slightly [55]. Specifically, wearables might be less accurate than ECG-based devices when participants are physically active versus resting (about 30% higher absolute error [56]). In our participants, audio recordings were coded for physical activity, which happened relatively rarely (in 2% of cases). However, future research should consider collecting objective movement data via accelerometry as an essential confounder and other covariates (for guidelines, see the study by Nelson et al [55]). Furthermore, future studies could consider integrating multiple physiological markers, such as blood pressure, cortisol, and heart rate variability, and leverage advanced sensor capabilities of wearables (eg, apple sensor kit [57]). This has the benefit of collecting data on markers sensitive to different features of everyday social contexts, for example, cortisol for socioevaluative situations and heart rate variability for prosocial behavior or compassion [58,59]. However, when doing so, it is vital to keep the inherent temporal dynamics of each system in mind. The appropriate sampling rate will differ between fast-acting systems, such as sympathetic activation as indexed by heart rate (seconds) versus hypothalamic-pituitary-adrenal activation as indexed by salivary cortisol (15-20 minutes) and needs to align with the psychosocial context under study [26].

### Heart Rate Linkage and Social Contexts and Exchanges

Our exploratory analysis found that heart rate linkage was stronger when raters coded partner conversations and couples self-reported interpersonal contact, compared with moments when raters did not code that partners were engaged in conversations or when couples did not self-report having had personal contact with each other. Findings dovetail with theoretical notions of the dyadic regulation connectivity model, which suggests that any changes in the dyadic system such as interpersonal environments trigger immediate and synchronized changes throughout the network of dynamically linked processes between partners, including their physiology [5]. Other studies have also shown increased physiological linkage when partners are in each other’s presence [52,60]. For example, research using a wristwatch to measure EDA in 40 young couples for 1 day showed that EDA linkage was significant only when couples self-reported being together but not apart [61]. When partners are engaged in conversation, they might follow the same narrative stimuli, which has been associated with increased heart rate linkage [62]. Our findings extend past research by focusing on moments when partners were actively interacting with each other instead of just being in physical proximity.

Heart rate linkage was also stronger when social support exchanges were self-reported by partners, as compared with moments when no social support exchanges were reported. Rater-coded social support was relatively infrequent (6%), preventing us from conducting exploratory analyses concerning its association with heart rate linkage. High physiological linkage has been associated with empathy, perspective-taking, positive interpersonal contexts such as feeling understood, appreciated or seeking help, and closeness to the partner [28,52,63]. A recent study investigating partners’ cardiovascular markers found significant physiological linkage in heart rate and heart rate variability in 27 young to middle-aged couples while discussing positive and negative aspects of their relationship in the laboratory [34]. In addition, a laboratory study found that synchronicity in younger couples’ skin conductance was higher during supportive touch when exposed to a pain stimulus, compared with the partner merely being present [64]. Our findings extend this research by indicating that heart rate fluctuations of couples managing a chronic health condition might link up during supportive, real-life interactions. Coherence between partners, for example, indicated by heart rate linkage, has been theorized to be central to dyadic regulation [5]. Yet, other studies demonstrated high physiological linkage in couples with marital strain, particularly regarding markers of the stress response system such as cortisol [65]. Potentially, parasympathetic nervous system markers (eg, respiratory sinus arrhythmia) might synchronize more during positive interpersonal contexts. In contrast, markers of the sympathetic nervous system (eg, EDA) might more likely become entrained during negative interpersonal contexts [28,29].

Future studies need to build on and replicate our findings using larger sample sizes, also looking at the underlying mechanisms and moderators of heart rate linkage, such as perspective taking, contagion of emotions, attachment style, relationship quality, and interdependent self-construal [28]. For example, is heart rate linkage stronger when partners try to take each other’s perspective and higher in couples with greater relationship satisfaction? Does lagged heart rate linkage accompany emotions (positive and negative) or stress being transferred from one partner to the other? [53] Such a study could use audio recordings to derive indices of vocal quality or pitch indexing emotional arousal such as the fundamental frequency [66]. It is also an open question whether stronger physiological heart rate linkage during social support exchanges is associated with more or less favorable outcomes of supportive interactions [67]. For example, increased skin conductance synchronicity was associated with less self-reported pain intensity during painful thermal stimulation when receiving supportive touch from the partner [64].

### Limitations and Future Research

Considering the sample size of 11 couples due to the impact of the COVID-19 pandemic on data collection, findings from this study need to be replicated to test generalizability. Furthermore, our sample comprised couples managing diabetes of mostly older age, long relationship duration, and relatively good diabetic control. Therefore, these couples could represent dyadic systems demonstrating reasonably effective dyadic regulation and thus higher heart rate linkage. It remains to be seen whether biopsychosocial dynamics differ by age and chronic disease type (eg, cancer [21]). In addition, we coded limited information about the nature of everyday social interactions between partners (social support occurrence, type of conversation), and interrater reliabilities were low for some coding categories. The unsatisfactory reliability of rater codings for conversation types prevented us from examining differences in heart rate linkage by type of conversation (eg, deep vs practical). Whether heart rate linkage is tied to the nature and content of partner conversations thus presents an important future direction.

Future studies could also investigate heart rate linkage in the context of support quality and types of support (emotional, instrumental). Given that previous literature has linked conflict to increased physiological linkage, it would also be important to study heart rate synchronization in the context of negative types of social exchanges managing chronic health conditions such as negative social control [68]. Future research should also examine other relevant constructs for couples managing disease, such as dyadic efficacy [69] or shared appraisals of the disease (“we-disease” [70]), and include measures of well-being (eg, happiness, meaning). Eventually, more advanced systems that collect multimodal data from couples managing chronic health conditions could learn when and how to intervene (eg, to nudge partners to provide support) to promote healthy lifestyle engagement and adaptive disease management [61,71]. Increasingly, researchers use machine learning algorithms to design just-in-time adaptive interventions [72,73], although they have seldom been applied in a dyadic context. These interventions can be delivered through digital platforms, making them widely accessible, particularly for individuals living in remote or underserved areas or those with transportation or mobility limitations.

### Conclusions

This study explored the feasibility of using commercial wearables to monitor the unique communication and support dynamics between romantic partners, particularly in managing a chronic health condition. First, the rater-coded audio recordings and self-report analysis provided valuable information about the occurrence of social support. Interestingly, while self-reports indicated that partners reported social support approximately half of the time, raters coded social support in only 6% of the recordings. This suggests a potential disparity between partners’ self-perception and external observation of supportive behaviors. Second, we demonstrated that couples exhibited small to moderate interconnections in heart rate fluctuations, indicating physiological linkage between partners. Heart rate linkage was stronger when partner conversations were coded, partners self-reported interpersonal contact, and partners self-reported social support exchanges. Findings provide initial evidence on contexts and behaviors that may influence physiological interconnectivity within couples. Overall, the use of wearables for continuous and unobtrusive collection of biopsychosocial data combined with self-report data in real-world settings holds great promise for enabling better and targeted support for individuals managing chronic health conditions and their partners.

## Appendix

Multimedia Appendix 1 Magnitude of cross-correlation by rater-coded and self-reported social context and exchange. The figure shows that cross-correlations tended to be higher in situations when raters coded that partners were engaged in conversations (A), when participants self-reported a partner interaction (B), and when participants self-reported a social support exchange (C).

## Abbreviations

DYMAND: DYadic MANagement of Diabetes
ECG: electrocardiogram
EDA: electrodermal activity
HbA_1c_: hemoglobin A_1c_
ICC: intraclass correlation
