# Supplementary figures and images for: Using Wearables to Study Biopsychosocial Dynamics in Couples Who Cope With a Chronic Health Condition: Ambulatory Assessment Study
Source: JMIR Mhealth Uhealth. 2024 Aug 5;12:e49576. doi: 10.2196/49576 (PMC11333870; doi:10.2196/49576)

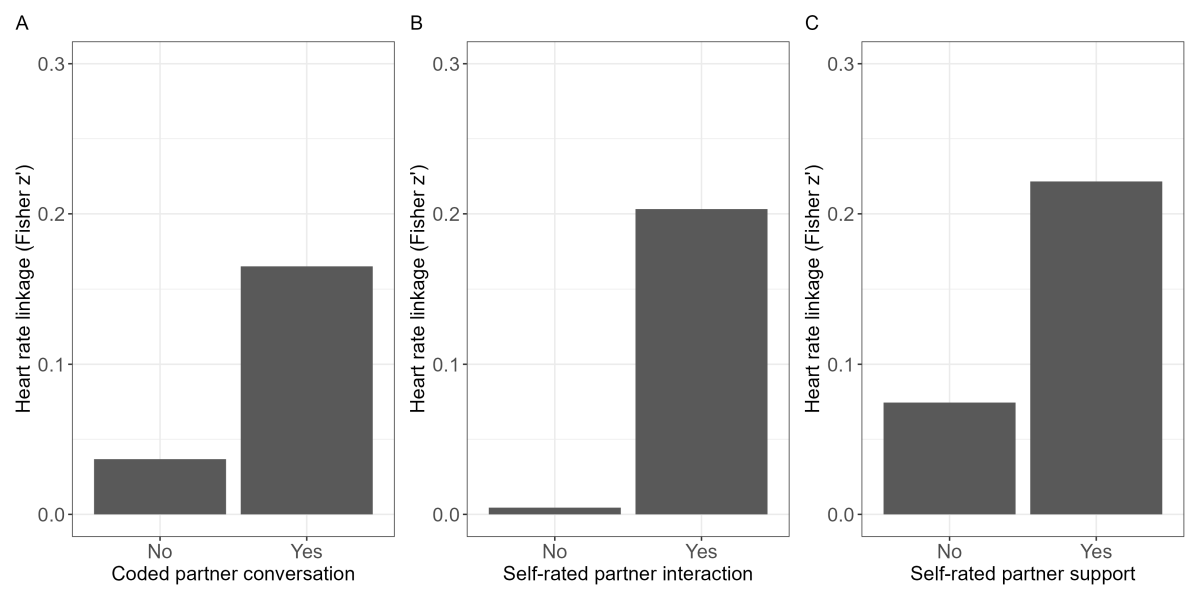

Supplement: Multimedia Appendix 1 [file mhealth_v12i1e49576_app1.png]
